# Balloon Pulmonary Angioplasty in Technically Operable and Technically Inoperable Chronic Thromboembolic Pulmonary Hypertension

**DOI:** 10.3390/jcm10051038

**Published:** 2021-03-03

**Authors:** Szymon Darocha, Aleksander Araszkiewicz, Marcin Kurzyna, Marta Banaszkiewicz, Stanisław Jankiewicz, Anna Dobosiewicz, Sylwia Sławek-Szmyt, Magdalena Janus, Maciej Grymuza, Arkadiusz Pietrasik, Tatiana Mularek-Kubzdela, Piotr Kędzierski, Radosław Pietura, Dariusz Zieliński, Andrzej Biederman, Maciej Lesiak, Adam Torbicki

**Affiliations:** 1 Department of Pulmonary Circulation, Thromboembolic Diseases and Cardiology, Centre of Postgraduate Medical Education, European Health Centre Otwock, 05-400 Otwock, Poland; szymon.darocha@ecz-otwock.pl (S.D.); marta.banaszkiewicz@ecz-otwock.pl (M.B.); annadobosiewicz@wp.pl (A.D.); piotr.kedzierski@ecz-otwock.pl (P.K.); adam.torbicki@ecz-otwock.pl (A.T.); 2 Department of Cardiology, Poznan University of Medical Sciences, 61-848 Poznan, Poland; aaraszkiewicz@interia.pl (A.A.); stachu145@wp.pl (S.J.); sylwiaslawek89@gmail.com (S.S.-S.); magdajanus@op.pl (M.J.); mgrymuza@vp.pl (M.G.); tatianamularek@wp.pl (T.M.-K.); maciej.lesiak@skpp.edu.pl (M.L.); 3 1st Department and Faculty of Cardiology, Medical University of Warsaw, 02-091 Warsaw, Poland; apietrasik@tlen.pl; 4 Department of Radiography, Medical University of Lublin, 00-635 Lublin, Poland; radoslawpietura@poczta.onet.pl; 5 Department of Cardiac Surgery, Medicover Hospital, 02-972 Warsaw, Poland; farok@wp.pl (D.Z.); abiederman@poczta.onet.pl (A.B.)

**Keywords:** balloon pulmonary angioplasty, chronic thromboembolic pulmonary hypertension, pulmonary endarterectomy, proximal-type CTEPH, distal-type CTEPH

## Abstract

Background: In this study, we aimed to assess the efficacy and safety of balloon pulmonary angioplasty (BPA) in patients with technically inoperable distal-type chronic thromboembolic pulmonary hypertension (d-CTEPH) and technically operable proximal-type disease (p-CTEPH) by analyzing the results of BPA treatment in two collaborating CTEPH referral centers. Methods and results: We assessed hemodynamic results, functional efficacy, complication and survival rate after BPA treatment in 70 CTEPH patients (median age 64 years; (interquartile range (IQR): 52–73 years)), of whom 16 (median age 73 years; (QR 62–82 years)) were in the p-CTEPH subgroup. Altogether, 377 BPA procedures were performed, resulting in significant (*p* < 0.001) improvement in mean pulmonary artery pressure (mPAP 48.6 ± 10 vs. 31.3 ± 8.6 mmHg), pulmonary vascular resistance (694 ± 296 vs. 333 ± 162 dynes*s*cm^−5^), six-minute walk test (365 ± 142 vs. 433 ± 120 metres) and N-terminal pro B-type natriuretic peptide (1307 (510–3294) vs. 206 (83–531) pg/mL). The rate of improvement did not differ between the sub-groups. Lung injury episodes and severe hemoptysis were similarly infrequent in d-CTEPH and p-CTEPH (6.4% vs. 5%; *p* = 0.55 and 1.0% vs. 2.5; *p* = 0.24, respectively). There was no significant difference between the sub-groups regarding survival (*p* = 0.53 by log-rank test). Conclusion: BPA may be beneficial in patients with p-CTEPH who cannot undergo pulmonary endarterectomy (PEA). Larger long-term studies are needed to better define the efficacy, safety, and optimal BPA procedural standards in this population.

## 1. Introduction

Pulmonary endarterectomy (PEA) remains the treatment of choice for patients suffering from operable chronic thromboembolic pulmonary hypertension (CTEPH) [1]. It is technically demanding, requires intermittent total circulatory arrest performed under deep hypothermia, but was found to be highly effective for both improving pulmonary hemodynamics and the otherwise poor survival expectancy in CTEPH [2,3]. Despite increasing the skills of PEA surgeons and the optimization of periprocedural care, some patients still cannot be operated on. The cause for this is either distal localization of thrombi, an unacceptably high risk of PEA due to comorbidities or the patient’s refusal to undergo complex surgery. Medical and interventional therapy, the latter consisting of balloon pulmonary angioplasty (BPA), have been successfully introduced for patients who are technically inoperable due to occlusions limited to surgically inaccessible distal arteries (d-CTEPH) [4].

Finding the optimal therapy for CTEPH patients is a challenging issue. Criteria for inoperability are mainly found in the anatomical distribution of pulmonary arterial lesions, but severe comorbidities also influence the decision for or against surgery. Following this, there is a group of technically inoperable patients (with only distal disease) and a (smaller) group of patients with technically operable disease, but incalculable risk for PEA. Obviously, assignment to the first group depends mainly on the surgeon’s expertise. There are no published data to indicate a threshold of surgical accessibility from preoperative imaging. Clinical experience indicates that both techniques, PEA and BPA, can access subsegmental disease and both are viable treatment options for disease confined to the segmental level. The degree of anatomical overlap between what disease is accessible to PEA and BPA is thought to be considerable, although there are no RCTs directly comparing both techniques. While there is no published evidence to support BPA as a treatment option in patients with potentially operable disease, it could still be justified based on the current understanding of CTEPH pathophysiology and particularly by the short life expectancy of patients not operated on, as reported earlier by our team and others [5,6,7]. Encouraged by our growing experience in BPA [8], we have started to use it also in patients with technically operable, proximal occlusions (p-CTEPH) who were not qualified for PEA surgery by multidisciplinary CTEPH team because of comorbidities or who refused to undergo such a complex procedure. In this study, we sought to assess the efficacy and safety of BPA treatment in the whole treated population as well as after dividing the population into sub-groups with technically inoperable and technically operable CTEPH.

## 2. Materials and Methods

### 2.1. Patient Selection

Between June 2013 and December 2018, a central multidisciplinary CTEPH team, which served both participating centers in management decisions and consisted of cardiac surgeons experienced in PEA, cardiologists experienced in pharmacotherapy of pulmonary hypertension (PH) and cardiologists experienced in balloon pulmonary angioplasty (BPA) analyzed 205 patients in terms of further treatment strategy. This selection was based on the anatomical location of thrombotic lesions and the risk of the PEA or BPA treatment, defined by the presence of comorbidities. The decision was established arbitrarily based on the experience of the surgical team and unfavorable factors related to the patients, like severe concomitant diseases, advanced age or frailty syndrome. Patients with p-CTEPH who were rejected from PEA by the CTEPH-team or who did not consent to the surgery were considered for BPA. The main contraindications to BPA were large central clots causing complete closure of the main or lobar branches, severe comorbidities limiting survival below six months (e.g., cancer), renal failure or hyperthyroidism restricting the possibility of using iodine contrast, and a lack of consent for the interventional treatment.

**Figure 1:**
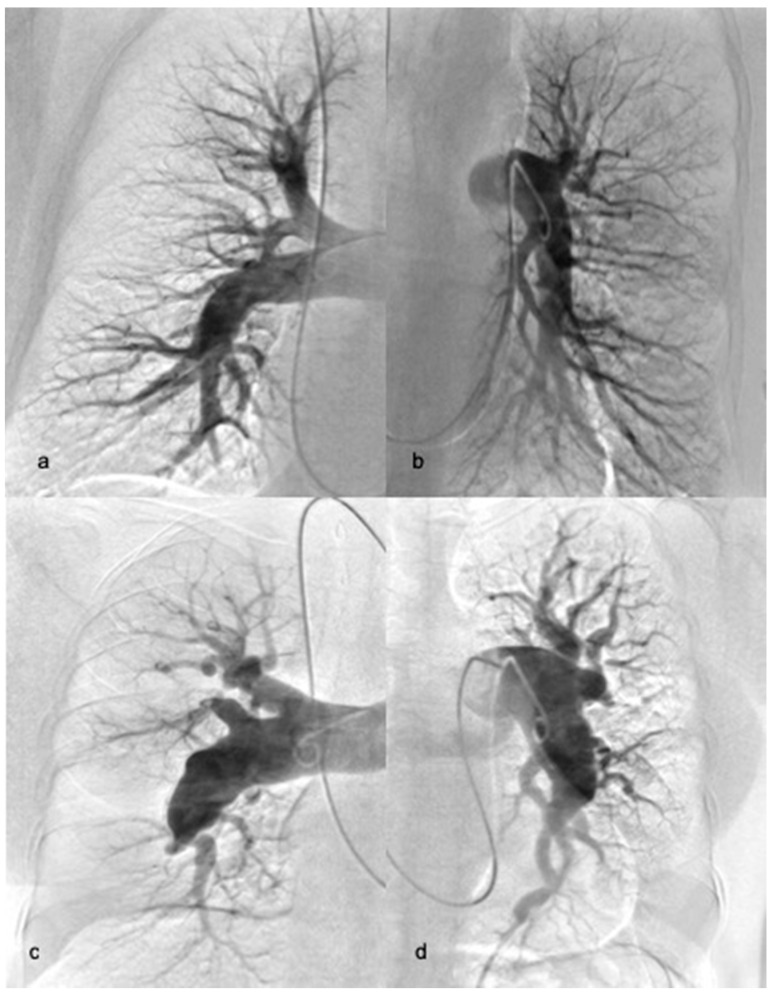
Example of right (**a**) and left (**b**) pulmonary angiography of patient with distal-type chronic thromboembolic pulmonary hypertension (CTEPH) and right (**c**) and left (**d**) pulmonary angiography of patient with proximal-type CTEPH.

Consequently, two sub-groups of non-operable patients were identified: patients with distal occlusions limited to the segmental and subsegmental level (d-CTEPH) (Figure 1a,b) and patients with proximal type CTEPH but who were rejected from surgery due to comorbidities and unfavourable risk-to-benefit ratios or those who refused to undergo surgery (p-CTEPH) (Figure 1c,d). At the time of the first BPA, most patients were on stable specific pharmacotherapy with riociguat, sildenafil, and/or treprostinil, according to current guidelines for non-operable CTEPH [9].

### 2.2. Diagnosis of CTEPH

CTEPH was diagnosed according to current guidelines, including right heart catheterization and imaging of pulmonary circulation consisting of at least two modalities such as selective pulmonary angiography, computed tomography or perfusion/ventilation scintigraphy, performed after at least 3 months of anticoagulation [9]. Haemodynamic parameters were measured with a Swan–Ganz catheter. Cardiac output (CO) was determined using the thermodilution method [10]. Arterial oxygen saturation was measured with pulse oximetry and mixed venous saturation was analyzed in blood samples taken from the pulmonary artery.

Functional parameters included the World Health Organization (WHO) functional class and a 6-min walk test (6MWT). Laboratory markers included N-terminal pro B-type natriuretic peptide (NT-proBNP) concentration, serum creatinine (SC) levels and glomerular filtration rate (eGFR), estimated using the modification of diet in renal disease (MDRD) equation. All hemodynamic, functional and biochemical parameters were measured in all the patients before the first BPA procedure and 2–4 months after treatment completion.

The study protocol was approved by the institutional Ethics Committees in both participating centers. All patients provided written informed consent for the procedures and for participation in the study.

### 2.3. Balloon Pulmonary Angioplasty

BPA was performed in both centers, according to the same protocol, as a series of staged procedures [11,12]. At least 24 h before the first procedure, patients discontinued chronic antithrombotic treatments with vitamin K antagonists or direct oral anticoagulants (DOAC) and received bridging therapy with low-molecular-weight heparin. In most cases, access was achieved through the right femoral vein. An intravenous bolus of unfractionated heparin (2000–5000 units) was given at the beginning of each procedure and repeated after each hour of the procedure in a dose of 1000–2000 units. An MP, JR4, JL4 or AL1 6-F guiding catheter (Launcher; Medtronic, Minneapolis, MN, USA) was inserted into the right or left pulmonary artery through a 90 cm 6F vascular sheath (Flexor; Cook, Bloomington, IN, USA) to achieve a good selective approach to the target vessel. Then, 0.014-inch guidewires (Cruiser; Biotronik, Bülach, Switzerland; Whisper MS; Abbott Vascular, Santa Clara, CA, USA, Sion Blue, Asahi, Japan) were passed through the lesions and inserted distally in the subsegmental artery. Subsequently, the target branches were dilated with multiple balloon inflations using semi-compliant balloon catheters with a size between 1.25 and 10 mm. The balloon diameter and length were adjusted to the type of lesion, the degree of stenosis in the pulmonary artery observed with angiography and the severity of PH. We used a modified BPA strategy [11] with the routine use of undersized balloon catheters of diameter of 2.0–3.0 mm at the initial phase of treatment and 1:1 sizing at the optimization phase when mean pulmonary artery pressure (mPAP) was below 35 mmHg, as proposed by the Okayama group [13]. After deflation of the balloon, contrast was injected into the treated vessel in order to evaluate the angiographic effect of the procedure or to detect possible vessel injury.

In special cases, when the nature or significance of a lesion was unclear, additional diagnostic tools were applied, such as intravascular ultrasound (IVUS), optical coherence tomography (OCT) [14], or measurement of the pressure gradient across the evaluated lesion [15]. After completing the procedure, each patient was transferred to the cardiac intensive care unit, where vital functions and potential complications were monitored for 24–48 h. The amount of contrast medium (CM) for a single BPA procedure and summarized radiation for the whole treatment cycle were recorded. BPA treatment was terminated when there were no treatable lesions in the pulmonary arteries or when the mPAP was ≤25 mm Hg.

### 2.4. Complications and Survival

Periprocedural complications, including pulmonary artery injury (perforation, dissection) or pulmonary lung injury (LI), were classified according to the classification proposed by Inami et al. [16]. Haemoptysis which occurred during BPA was recognised as severe when blood volume exceeded 50 mL. The contrast-induced nephropathy (CIN) was defined as an increase in SC ≥ 25% or an absolute increase ≥ 0.5 mg/dL compared to the baseline value 48–72 h after exposure to CM. During the follow-up period patients were under the care of the center performing BPA, and long-term complications and survival were analyzed.

### 2.5. Statistical Analysis

Nominal variables are presented as numbers and percentage values. Continuous variables with normal distribution are presented as mean and standard deviation (SD). Variables with a distribution different from a normal distribution are presented as median and interquartile range (IQR). Student’s *t*-test or the Wilcoxon test (depending on the distribution of the analyzed variable, assessed with the Shapiro–Wilk test) was used to compare the continuous variables obtained from the data before and after BPA. The Mann U test and the Fisher exact test were used for comparison between groups for continuous and nominal independent variables. The proportion of patients who survived was estimated using the Kaplan–Meier method with the date of diagnostic angiography as the starting point. The survival rate in both groups with different locations of the thrombi was compared using the log-rank. All statistical analyses were performed using STATISTICA 13 software. A *p*-value < 0.05 was considered statistically significant.

## 3. Results

### 3.1. Demographic and Procedural Parameters

**Figure 2:**
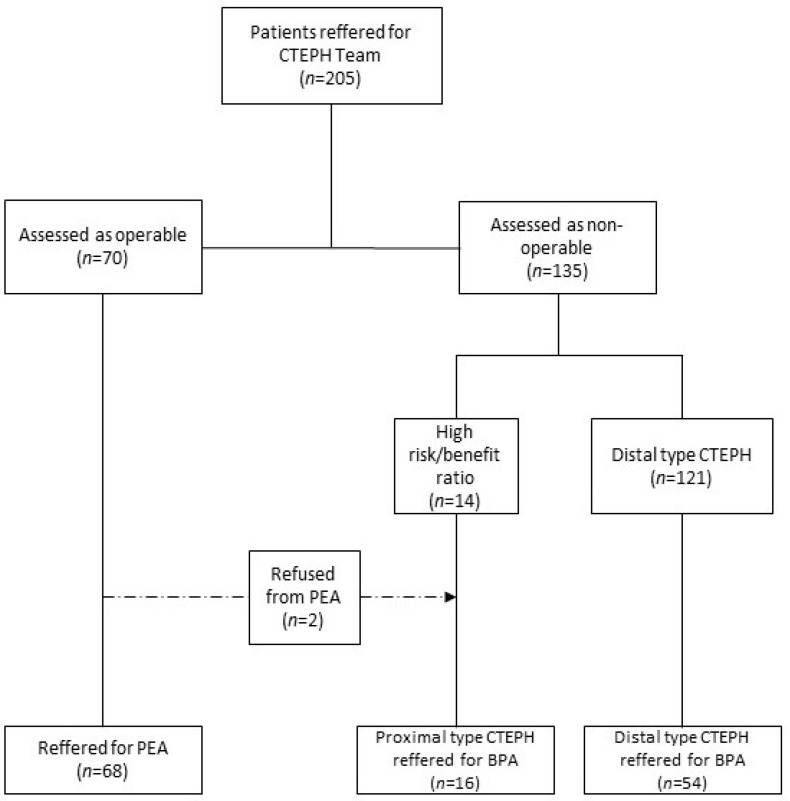
Flow chart presenting distribution of patients with CTEPH qualified to proper therapeutic strategies. BPA—balloon pulmonary angioplasty; PEA—pulmonary endarterectomy.

Among 205 patients evaluated by CTEPH team, 68 patients (33%) were assessed as technically operable and 137 (67%) were deemed inoperable, of which 70 (51%) patients were assessed as candidates for BPA and 67 (49%) patients were qualified for medical therapy only. Out of the 70 non-operable patients, 54 individuals (77.1%) were inoperable for anatomical reasons (d-CTEPH) and 14 (22.9%) were excluded from PEA because of comorbidities (p-CTEPH) or patients’ refusal. Patients’ distribution is presented in Figure 2.

Seventy patients (53% female) with inoperable CTEPH underwent a total of 377 BPA sessions (mean 5.4 ± 2.4 sessions per patient). A total of 297 BPA sessions were performed in the first group (d-CTEPH) and 80 procedures in the latter group (p-CTEPH). The clinical and procedural characteristics of the studied groups, including medical treatment before BPA, are shown in Table 1.

### 3.2. Subpopulation of Proximal CTEPH

Characteristics of patients with p-CTEPH, including age, main comorbidities and lesion position in pulmonary angiography according to the San Diego classification are presented in Supplementary Table S1 [17]. Patients from the p-CTEPH group were significantly older and more likely to suffer from chronic kidney disease. Two subjects from this group (BK, MP) were qualified for PEA but finally refused surgery and were referred for rescue BPA. Among many concomitant diseases, chronic respiratory insufficiency (SB, JI, HD, KM, WM, KM, ZS), and obesity with BMI > 35 kg/m2 (JI, MP, KM, HB, KM) were the main factors that decided the unfavourable risk/benefit ratio for PEA. In three patients (JG, JB, PJ) severe coronary artery disease with no option for revascularization, and in one case (ZP) severe aneurysm in the descending aorta was calculated as increasing the risk of PEA to an unfavourable level. In an 83-year-old patient (SG), an unfavourable risk-to-benefit ratio was estimated because of frailty syndrome and persistent immobility. P-CTEPH patients underwent 80 BPA procedures. Selective angiography revealed typical lesions for CTEPH, mainly subtotal occlusions, webs and bands, and ring-like stenoses. In the p-CTEPH group, BPA procedures initiated from coexisting distal lesions are considered technically easier to dilate, to achieve a significant reduction in mPAP (preferably < 35 mm Hg), and to reduce the risk of reperfusion injury. Subsequently, subtotal occlusions in the proximal segments of the arteries were treated. Treatment of the pouch lesions was mainly avoided with BPA. The maximum diameter of the balloon catheter used for the treatment of those lesions was similar to that in d-CTEPH. Supplementary Table S2 presents details of the BPA treatment in the two subpopulations.

### 3.3. Treatment Response

A total of 2379 lesions were dilated. The median number of pulmonary segments targeted in a single procedure was six (interquartile range 4–8). The target vessel distribution is presented in Supplementary Table S2. IVUS was used in 139 sessions (37%), OCT in 14 sessions (3.7%), and a pressure gradient measurement was used in 59 sessions (16%).

The effects of BPA on hemodynamic, laboratory parameters and functional capacity are presented in Table 2.

The main hemodynamic parameters showed significant improvement in both sub-groups in terms of mean pulmonary artery pressure (mPAP) and pulmonary vascular resistance (PVR), whereas cardiac index (CI), mean right atrial pressure (mRAP) and arterial oxygen saturation improved only in the d-CTEPH group. Pulmonary capillary wedge pressure (PCWP) and systemic blood pressure were significantly higher after BPA in the p-CTEPH group in comparison to the d-CTEPH group (Table 3).

There was also a significant improvement in functional capacity, measured as WHO functional class (WHO FC), in both studied groups. However, in the p-CTEPH group there was no significant improvement in 6MWT. In contrast with this result, the d-CTEPH group presented a 15.2% (*p* < 0.001) increase in 6MWT distance. The NT-proBNP serum level was reduced significantly and similarly in both groups.

### 3.4. Complications

The data regarding complications are presented in Supplementary Table S3. Out of 377 BPA sessions, LI was observed in 23 procedures (6.1%). LI appeared in 19 sessions (6.4%) of 297 procedures in d-CTEPH and in four sessions (5%) of p-CTEPH (*p* = NS). Severe hemoptysis was observed in three (1%) cases in d-CTEPH and two procedures (2.5%) in p-CTEPH. In one patient, an allergic reaction (urticaria and Quincke’s edema) was observed during the procedure. The mean contrast medium volume per procedure was higher in d-CTEPH; however, this did not cause a more frequent occurrence of CIN. The radiation dose did not differ significantly among studied groups.

### 3.5. Follow-Up

In total, six patients died (8.6%) during the observation period (median 36 months, IQR 23–48 months). One death (F, 70-year-old; d-CTEPH) occurred in the periprocedural period in a patient undergoing the third BPA session, in whom the two earlier BPA procedures did not result in hemodynamic and clinical improvement. The cause of death was severe failure of the right ventricle without any apparent signs of periprocedural lung injury. Four patients died during the treatment cycle, but without a direct relationship to the procedures performed. In detail, two of four died suddenly at home (M, 66-year-old, d-CTEPH; and F, 81-year-old, p-CTEPH) 7 weeks after the fourth BPA session, one of four (M, 69-year-old, p-CTEPH) died of pneumonia and one (F, 64-year-old, d-CTEPH) due to acute leukaemia 3 months after the second and fourth BPA procedures, respectively. In addition, one patient (M, 70-year-old, d-CTEPH) died 5 months after completing BPA treatment due to an infectious exacerbation of severe COPD. Overall survival rates at 1, 3 and 5 years were 97.1%, 92.0% and 88.4%, respectively (95%CIs–94.3%–99.9%, 85.1–98.9% and 71.9–98%, respectively). There was no significant difference between p-CTEPH and d–CTEPH groups regarding survival. Cumulative survival rates at 1, 3 and 5 years in the d–CTEPH group were 96.2%, 91.5% and 91.5%, whereas in the p–CTEPH group they were 93.8%, 93.8% and 80.4%, respectively (*p* = 0.53 by log-rank test).

## 4. Discussion

In this study, we have reported the joint results of BPA from our two collaborating PH referral centers. Both submitted their CTEPH patients for treatment decisions to the same central national interdisciplinary CTEPH team and conformed to identical protocols for BPA. Within the scope of effectiveness, our results are comparable to other European series published by French and German centers [18,19], but are still slightly worse than the results achieved in Japan [20,21]. In our opinion, this is due not only to lower experience in the field of BPA, but is related to higher baseline values of pulmonary artery pressure and pulmonary vascular resistance in patients with CTEPH in the studied population [22]. Hence, interventional treatment of CTEPH may be more challenging in European centers, because higher initial pressures may indicate more severe damage to the pulmonary microcirculation. We confirmed a good safety of BPA using the refined strategy based on a two-step approach with many undersized dilatations at the beginning of therapy and complete revascularization with adequately sized balloon catheters in the optimization phase, as we previously reported [11].

PEA is considered the standard treatment for CTEPH patients [3,23,24] and can provide a dramatic improvement in symptoms and restore life expectancy to normal. There is profound evidence that surgery in operable CTEPH patients leads to normalization of pulmonary hemodynamics in the majority of patients, with excellent long-term prognosis. At this moment, BPA should not be considered as a replacement for PEA in operable CTEPH. The standard surgical technique for PEA has not changed significantly in the last years and is still based on the principles established by the San Diego group [2]. The procedure involves the removal of fibrous obstructive tissue from pulmonary arteries during circulatory arrest under deep hypothermia. Given the complexity of PEA, the decision of operability depends not only on the location of intravascular lesions but also on comorbidities, functional parameters, hemodynamics and the patient’s acceptance of the risk of surgery. Over the last years, the most significant surgical progress has been in redefinition of the distal limits of PEA. In experienced centers, patients with very distal chronic thromboembolism can undergo PEA with good results. The progress in diagnostics and increasing surgical experience have contributed to this process. Consequently, the current intraoperative San Diego classification better mirrors the present surgical approach [17]. However, it is worth noting that the attempt to use the San Diego classification to assess angiography as a key imaging method in qualifying patients for PEA may underestimate the size of pulmonary arterial thrombi. Thus, surgically accessible patients may be classified as technically inoperable by imaging. In the prospective registry containing PEA outcome data from 27 expert centers, 427 (62.9%) from 679 consecutive patients diagnosed with CTEPH were considered operable on the basis of the surgeon’s assessment. Finally, 386 patients (56.8%) underwent surgery, including 13 patients documented as inoperable; 38 operable patients refused the procedure, and seven patients died before surgery. The in-hospital mortality rate after PEA was 4.7%, despite some centers having low number of procedures performed yearly and a low level of experience. The expert center in PEA was defined to perform > 50 PEA annually. It is worth noting that the center’s expertise, as defined by the number of PEA procedures performed per year, was not a risk factor for mortality [25], but there was a clear trend. A larger series from an experienced center reported a mortality of 2.2% for a cohort of last 500 patients in comparison to 5.2% in-hospital mortality for the cohort of the first 1000 patients (*p* < 0.01). [3]. At our center, the survival benefit of PEA was noted despite an average 9.1% surgical in-hospital mortality rate calculated between 1998 and 2008 [7]. Such a level of risk is compatible with previous reports from various centers, where it ranged from 2.2% to 16% [3,25,26,27,28,29].

In parallel, BPA has emerged as a new therapeutic technique in patients with inoperable CTEPH. With the initiation of the BPA program, the proportion of patients undergoing surgical and interventional treatment is changing. Amsallem et al., in a study based on the French National Reference Centre CTEPH prospective registry, showed the influence of the initiation of the BPA programme on the PEA programme [30]. Although the total number of patients undergoing PEA remained stable between the pre-BPA era and the BPA era, the respective proportion of patients operated on among the total cohort of patients with CTEPH decreased (*p* < 0.01). Previously, patients with central-type CTEPH have been excluded from BPA due to concerns regarding the effectiveness and safety of this treatment applied to proximal dilated arteries filled with thick fibrotic organized lesions. Even if it is locally successful, it could result in extensive pulmonary edema, which would lead to morbidity and mortality [4,31]. With experience increasing rapidly in high-volume BPA centers, this method emerged naturally as a potential therapeutic option for patients with CTEPH who remained non-operated despite the important contribution of proximal lesions. Evidence, however, was lacking, if not counting a single case report by Ishiguro et al., who in 2013 first demonstrated that staged revascularization by BPA can be an effective method in the treatment of central-type CTEPH [32]. Similarly, a smaller study was published this year by Minatsuki et al. reporting on Japanese patients suffering from CTEPH. They enrolled 33 patients with “BPA-suitable CTEPH” and 10 patients with “BPA-unsuitable CTEPH” [33]. In all patients, they reported improvement in hemodynamic and functional parameters, with no significant differences between the groups. Importantly, the rate of complications, especially pulmonary bleeding, did not differ between the groups. However, more sessions were required in the BPA-unsuitable group (BPA-suitable group—four sessions vs. BPA-unsuitable group—six sessions). We found that the effectiveness and safety of BPA were similar in the groups with either distal- or proximal-type CTEPH. According to our best knowledge, this is first report from non-Japanese centers. Initially, the differences between the groups were small and related to age and the more frequent occurrence of chronic kidney disease in the group of technically inoperable patients. In these patients, we used less contrast, without observing a more frequent development of nephropathy, which also confirms our previous observations [34]. Our study revealed significant improvements in both groups in terms of main hemodynamic parameters, like mPAP and PVR, without a change in CI and oxygenation in the p-CTEPH group. Significant improvement in both groups was also observed in terms of functional capacity and biochemical parameters. The 1-year and 3-year survival in the p-CTEPH group was achieved at the level of 94%, which contrasts with the 79% survival rate of the CTEPH patients treated solely with medical therapy reported previously [35].

The two groups do not differ in the maximum size of the balloon catheter used, which shows that even in d-CTEPH, single lesions in larger vessels may coexist and may require treatment. Despite this, we did not observe significant differences in complication rates between the two groups. The prerequisite for obtaining a good hemodynamic effect is maximum extensive revascularization [36], which is why we strove to perform procedures within all lung segments. However, this is associated with the need to carry out more procedures and the patients’ exposure to radiation and contrast, which is not found in PEA. Despite this, we found BPA to be a very promising option in the treatment of proximal CTEPH.

## 5. Conclusions

BPA may be beneficial in patients with proximal technically operable CTEPH who cannot undergo PEA. Larger long-term studies are needed to better define efficacy, safety, and optimal BPA procedural standards in this population.

## 6. Limitations

Our study has several limitations. The p-CTEPH group was relatively small, but still larger than the only series ever previously published. Some of the improvements observed in both sub-groups could be assigned to concomitant medical therapy. However, at the time of starting BPA, most of the effects of medical treatment introduced several months earlier would have already occurred. We analyzed data from two centers, each having had their patients evaluated for optimal management by the same central CTEPH team and performing BPA using the same technique. Finally, our follow-up is relatively short, but Japanese data showed that in long-term observation, PAP and PVR values do not increase after successful BPA [21], and low PAP at the end of the treatment provides good long-term prognosis [37,38]. Although our patients with p-CTEPH may have higher long-term mortality rates, this may be due to their higher age and not necessarily the ineffectiveness of BPA treatment.

## Figures and Tables

**Table 1:** Clinical characteristics of the studied population.

	All Patients (*n* = 70)	d-CTEPH Group (*n* = 54)	p-CTEPH Group (*n* = 16)	*p*
Age (years)	64.5 (52–73)	62.5 (48–70)	73 (62–82)	0.008
Females (%)	37 (53%)	29 (54%)	8 (50%)	0.5
BMI (kg/m^2^)	25.8 (23.3–28.5)	25.5 (23.4–28.1)	26.4 (23.0–33.3)	0.35
History of PE	52 (74%)	41 (76%)	11 (69%)	0.39
History of DVT	24 (34%)	17 (31%)	7 (44%)	0.27
**Comorbidities**				
Coronary heart disease	13 (19%)	8 (15%)	5 (31%)	0.13
Systemic hypertension	33 (47%)	24 (44%)	9 (56%)	0.29
Type II diabetes	11 (16%)	9 (17%)	2 (13%)	0.51
COPD	6 (9%)	4 (7%)	2 (13%)	0.42
Chronic kidney disease (eGFR < 60 mL/min)	12 (17%)	6 (11%)	6 (38%)	0.02
Atrial fibrillation/flutter	12 (17%)	9 (17%)	3 (19%)	0.55
Pacemaker leads or ventriculoatrial shunts	6 (9%)	6 (11%)	0	0.19
**Concomitant therapy**				
Sildenafil	20 (29%)	15 (28%)	5 (31%)	0.51
Riociguat	42 (60%)	31 (57%)	11 (69%)	0.30
Prostanoids	1 (1%)	1 (2%)	0	-
PAH-like monotherapy	61 (87%)	45 (83%)	16 (100%)	0.08
PAH-like combination therapy	1 (1%)	1(2%)	0	-
VKA	22 (31%)	17 (31%)	5 (31%)	0.9
LMWH	8 (11%)	5 (9%)	3 (19%)	0.26
DOAC	40 (57%)	32 (59%)	8 (50%)	0.35

BMI—body mass index; PE—pulmonary embolism; DVT—deep vein thrombosis; COPD—chronic obstructive pulmonary disease; eGFR—estimated glomerular filtration rate; PAH—pulmonary arterial hypertension; VKA—vitamin K antagonists; LMWH–low molecular weight heparin; DOAC—direct oral anticoagulants.

**Table 2:** Clinical and hemodynamic effect of BPA in all patients.

	All Patients (*n* = 70)		
	Before	*p*	After
HR (bpm)	77 ± 15	<0.001	68 ± 12
BP systolic (mmHg)	129 ± 26	0.18	126 ± 20
BP diastolic (mmHg)	76 ± 14	0.002	71 ± 10
WHO class (I–II/III–IV)	20%/80%	<0.001	77%/23%
6mWT (m)	365 ± 142	<0.001	433 ± 120
NT-pro-BNP (pg/mL)			
(median)	1307		206
(IQR)(IQR)	(510–3294)	<0.001	(83–531)
mRAP (mmHg)	9.1 ± 4.4	<0.001	5.6 ± 3.2
PAPs (mmHg)	81.6 ± 18.3	<0.001	53.8 ± 16.4
PAPd (mmHg)	28.5 ± 7.4	<0.001	17.0 ± 5.8
PAPm (mmHg) (median) (IQR)	48.6 ± 10.0 48 (41–55)	<0.001	31.3 ± 8.6 30 (26–36)
PCWP (mmHg)	9.9 ± 2.7	0.99	10.0 ± 3.6
CO (L/min)	4.96 ± 1.58	0.04	5.44 ± 1.45
CI (L/min*m^2^)	2.75 ± 0.78	0.03	2.95 ± 0.78
SV (ml)	65 ± 20	<0.001	80 ± 20
Art Sat. O_2_(%)	93.4 ± 3.3	0.01	94.9 ± 3.8
MV Sat.O_2_(%)	65.2 ± 6.8	<0.001	72.2 ± 6.3
PVR (dynes*s*cm^−5^) (median) (IQR)	694 ± 296 643 (465–892)	<0.001	333 ± 162 282 (229–396)
CPa (mL/mmHg)	1.40 ± 0.76	<0.001	2.39 ± 0.88
Absolute change of mPAP(mmHg)	−16.8 ± 9.9		
% decrease of mPAP (%)	−34 ± 17		
% decrease of PVR (%)	−46 ± 22		

HR–heart rate; BP–blood pressure; WHO–World Health Organisation; 6mWT–6 min walk test; NT-proBNP–N-terminal probrain natriuretic peptide; mRAP–mean right atrial pressure; PAPs–systolic pulmonary artery pressure; PAPd–diastolic pulmonary artery pressure; PAPm–mean pulmonary artery pressure; PCWP, pulmonary capillary wedge pressure; CO–cardiac output; CI–cardiac index; SV–stroke volume; Art.SatO2–arterial oxygen saturation; MvSatO2–mixed venous oxygen saturation; PVR–pulmonary vascular resistance; CPa–pulmonary artery compliance. Data are presented as means ± standard deviations or median and (interquartile range).

**Table 3:** Clinical and hemodynamic effect of BPA in anatomically inoperable patients (d-CTEPH) and inoperable patients because of comorbidities or patients’ refusal (p-CTEPH).

	Anatomically Inoperable (d-CTEPH Group) (*n* = 54)			Inoperable Because of Comorbiditie (p-CTEPH Group) (*n* = 16)		
	Before	*p*	After	Before	*p*	After
HR (bpm)	78 ± 15	<0.001	69 ± 13	75 ± 16	0.04	67 ± 9
BP systolic (mmHg)	126 ± 24	0.24	121 ± 16 **	140 ± 30	0.55	142 ± 25 **
BP diastolic (mmHg)	76 ± 14	<0.001	70 ± 10 *	77 ± 14	0.88	77 ± 9 *
WHO class (I–II/III–IV)	20%/80%	<0.001	77%/23%	19%/81%	0.02	79%/21%
6mWT (m)	384 ± 131	<0.001	453 ± 115 *	300 + 164	0.11	355 ± 112 *
NT-pro-BNP (pg/mL) (median) (IQR)	1380 (330–3294)	<0.001	157 (77–448)	1288 (754–2870)	<0.001	255 (203–897)
mRAP (mmHg)	9.4 ± 4.7	<0.001	5.6 ± 3.4	8.4 ± 3.3	0.05	5.6 ± 2.5
PAPs (mmHg)	82.1 ± 18.3	<0.001	54.1 ± 17.7	80.0 ± 18.5	0.001	52.9 ± 10.8
PAPd (mmHg)	29.3±6.7	<0.001	25.8 ± 9.1 *	25.8 ± 9.1	0.02	16.9 ± 3.9 *
PAPm (mmHg) (median) (IQR)	49.0 ± 10.0 49 (43–55)	<0.001	31.3 ± 9.3 31 (25–36)	47.1 ± 10.1 46 (41–52)	0.001	31.2 ± 5.6 29 (27–36)
PCWP (mmHg)	9.7 ± 2.8	0.65	9.4 ± 3.3 *	10.4 ± 2.3	0.44	12.1 ± 4.2 *
CO (L/min)	4.94 ± 1.65	0.06	5.43 ± 1.53	5.03 ± 1.39	0.43	5.47 ± 1.17
CI (L/min*m^2^)	2.72 ± 0.78	0.04	2.95 ± 0.80	2.88 ± 0.78	0.50	2.96 ± 0.67
SV (ml)	65 ± 21	<0.001	79 ± 20	68 ± 18	0.01	83 ± 18
Art Sat. O_2_(%)	93.8 ± 3.1	0.01	95.2 ± 3.8	93.7 ± 3.1	0.45	93.9 ± 3.6
MV Sat.O_2_(%)	65.7 ± 6.7	<0.001	72.5 ± 6.4	63.6 ± 7.0	0.01	70.9 ± 6.1
PVR (dynes*s*cm^−5^) (median) (IQR)	713 ± 305 683 (479–935)	<0.001	345 ± 176 286 (223–420)	628 ± 263 556 (429–862)	0.001	288 ± 87 278 (245–303)
CPa (mL/mmHg)	1.38 ± 0.72	<0.001	2.39 ± 0.93	1.50 ± 0.89	0.01	2.39 ± 0.61
Absolute change of mPAP(mmHg)	−17.1 ± 9.4			−15.9 ± 12.0		
% decrease of mPAP (%)	−35 ± 16			−31 ± 19		
% decrease of PVR (%)	−46 ± 23			−46 ± 19		

HR–heart rate; BP–blood pressure; WHO–World Health Organisation; 6mWT–6 min walk test; NT-proBNP–N-terminal probrain natriuretic peptide; mRAP–mean right atrial pressure; PAPs–systolic pulmonary artery pressure; PAPd–diastolic pulmonary artery pressure; PAPm–mean pulmonary artery pressure; PCWP–pulmonary capillary wedge pressure; CO–cardiac output; CI–cardiac index; SV–stroke volume; Art.SatO2–arterial oxygen saturation; MvSatO2–mixed venous oxygen saturation; PVR–pulmonary vascular resistance; CPa–pulmonary artery compliance. Data are presented as means ± standard deviations or median and (interquartile range); * *p* < 0.05; ** *p* < 0.01 for comparisons between anatomically inoperable and inoperable because of comorbidities.

## Supplementary Materials

The following are available online at https://www.mdpi.com/2077-0383/10/5/1038/s1, Table S1: Characteristics of patients with proximal type CTEPH rejected from PEA—age, lesions position according to San Diego classification [17] assessed with pulmonary angiography, main comorbidities; Table S2: Lung segments treated with BPA and balloon catheter size used; Table S3: Risk related to BPA treatment—procedural complications, contrast medium usage and radiation exposure.
