# Supplementary material for: Balloon Pulmonary Angioplasty in Technically Operable and Technically Inoperable Chronic Thromboembolic Pulmonary Hypertension
Source: J Clin Med. 2021 Mar 3;10(5):1038. doi: 10.3390/jcm10051038 (PMC7959461; doi:10.3390/jcm10051038)
Supplement: Supplementary file 1 [file jcm-10-01038-s001.pdf]

Supplementary Table S1. Characteristics of patients with proximal type CTEPH rejected from PEA—age, lesions position according to San Diego classification [17] assessed with pulmonary angiography, main comorbidities.

Patient	Age	Lesions position according to San Diego classification (right/left)	Reason for rejection from PEA	Main comorbidities
BK	54	II/II	Patient's refusal	Hypothyroidism
SB	81	I/II	Unfavourable risk-to-benefit ratio	prostatic cancer, COPD (C class according to GOLD), pulmonary trunk aneurysm (47mm),
ZP	72	III/II	Unfavourable risk-to-benefit ratio	chronic kidney disease (stage G3b), ascending aortic aneurysm, arterial hypertension
JI	85	II/III	Unfavourable risk-to-benefit ratio	COPD with chronic respiratory insufficiency, obesity (BMI 40,3 kg/m2), multivessel coronary artery disease
JG	83	I/III	Unfavourable risk-to-benefit ratio	Multivessel coronary artery disease, atrial fibrillation, chronic kidney disease (stage G3a)
MP	60	I/II	Patient's refusal	Chronic kidney disease (stage G3a), obesity (BMI 42,7 kg/m2), psoriatic arthritis, hypothyroidism
HD	69	II/III	Unfavourable risk-to-benefit ratio	Multivessel coronary artery disease, history of myocardial infarction, COPD, cardiac pacing system, arterial hypertension
PJ	69	II/III	Unfavourable risk-to-benefit ratio	Dementia syndrome, coronary artery disease, atrial fibrillation, arterial hypertension
KM	78	I/III	Unfavourable risk-to-benefit ratio	History of colorectal cancer, chronic kidney disease (stage G3a), diabetes mellitus type 2, COPD, obesity (BMI: 37,7 kg/m2), severe aortic valve stenosis
SG	83	II/II	Unfavourable risk-to-benefit ratio	chronic kidney disease (stage G3a), atrial fibrillation, poor mobility
HB	62	II/III	Unfavourable risk-to-benefit ratio	chronic kidney disease (stage G3a), arterial hypertension, diabetes mellitus type 2, history of GI bleeding, obesity (BMI: 35,6 kg/m2)
WM	83	II/I	Unfavourable risk-to-benefit ratio	Coronary artery disease, COPD
ZS	77	II/III	Unfavourable risk-to-benefit ratio	Multivessel coronary artery disease, COPD

JZ	80	III/II	Unfavourable risk-to-benefit ratio	Arterial hypertension, diabetes mellitus type 2, chronic kidney disease (stage G3a)
JB	67	II/II	Unfavourable risk-to-benefit ratio	Multivessel coronary artery disease, CABG in history, history of colon cancer treated surgically, arterial hypertension, hyperthyroidism, atrial fibrillation
KM	66	III/II	Unfavourable risk-to-benefit ratio	Arterial hypertension, COPD, obesity (BMI: 36,2 kg/m2)

BMI – body mass index; COPD – chronic obstructive pulmonary disease; CABG – coronary artery bypass graft′ GOLD – global Initiative for Chronic Obstrucitve Lung Disease; GI – gastrointestinal tract; PEA – pulmonary endarterectomy

Supplementary Table S2: Lung segments treated with BPA and balloon catheter size used .

	All patients (n=70)	d-CTEPH group (n=54)	p-CTEPH group (n=16)	p
**Left lung**				
Maximal diameter of balloon catheter (median; IQR) [mm]	4.0 [3.5 - 6.0]	4.5 [3.5 - 6.0]	3.5 [2.5 - 6.0]	0.29
LA 1+2	38 (54%)	31 (55%)	7 (50%)	0.95
LA3	21 (30%)	18 (32%)	3 (21%)	0.65
LA4	35 (50%)	29 (52%)	6 (43%)	0.77
LA5	42 (60%)	33 (59%)	9 (64%)	0.95
LA6	37 (53%)	29(52%)	8 (57%)	0.90
LA8	53 (76%)	45 (80%)	8 (57%)	0.14
LA9	56 (80%)	48 (86%)	8 (57%)	0.04
LA10	53 (76%)	46 (82%)	7 (50%)	0.03
**Right lung**				
Maximal diameter of balloon catheter (median; IQR) [mm]	5.0 [4.0 - 6.0]	5.0 [4.0 - 6.0]	5.0[4.0 - 6.0]	0.99
RA1	48 (69%)	38 (68%)	10 (71%)	0.95
RA2	48 (69%)	38 (68%)	10 (71%)	0.95
RA3	38 (54%)	31 (55%)	7 (50%)	0.95
RA4	40 (57%)	34 (61%)	6 (43%)	0.36
RA5	46 (66%)	40 (71%)	6 (43%)	0.09
RA6	39 (56%)	27 (48%)	12 (86%)	0.03
RA7	27 (39%)	25 (45%)	2 (14%)	0.07
RA8	59 (84%)	48 (86%)	11 (79%)	0.80
RA9	53 (76%)	42 (75%)	11 (79%)	0.94
RA10	57 (81%)	46 (82%)	11 (79%)	0.94

Supplementary Table S3. Risk related to BPA treatment – procedural complications, contrast medium usage and radiation exposure

	**All patients** (377 *procedures*)	**d-CTEPH group** *(297 procedures)*	**p-CTEPH group** *(80 procedures)*	**p**
Procedure related death	1 (0.3%)	1 (0.4%)	0	NA
Lung injury (per procedure)	23 (6.1%)	19 (6.4%)	4 (5%)	0.44
mild/moderate	21 (5.6%)	17 (5.7%)	4 (5%)	0.53
severe	2 (0.5%)	2 (0.7%)	0	NA
Hemoptysis (per procedure)	23 (6.1%)	20 (6.7%)	3 (3.8%)	0.24
blood volume < 50 ml	18 (4.8%)	17 (5.7%)	1 (1.3%)	0.07
blood volume > 50 ml	5 (1.3)	3 (1.0%)	2 (2.5%)	0.29
PA dissection (per procedure)	7 (0.3%)	5 (1.7%)	2 (2.5%)	0.46
PA perforation (per procedure)	22 (5.8%)	18 (6.1%)	4 (5%)	0.45
Mean contrast medium (per procedure) [ml]	262 ± 53	270 ± 52	237 ± 52	0.03
Contrast induced nephropathy (per procedure)	8 (2.1%)	6 (2.0%)	2 (2.5%)	0.53
Radiation quantity – air KERMA (per patient's treatment cycle) [mGy]	4675 [2404-6346]	4801 [2768-6308]	3320 [2155-7033]	0.59

PA - pulmonary artery
